# Trophic Interactions of Infant Bifidobacteria and *Eubacterium hallii* during L-Fucose and Fucosyllactose Degradation

**DOI:** 10.3389/fmicb.2017.00095

**Published:** 2017-01-30

**Authors:** Clarissa Schwab, Hans-Joachim Ruscheweyh, Vera Bunesova, Van Thanh Pham, Niko Beerenwinkel, Christophe Lacroix

**Affiliations:** ^1^Laboratory of Food Biotechnology, Department of Health Sciences and Technology, ETH ZurichZurich, Switzerland; ^2^Department of Biosystems Science and Engineering, ETH ZurichBasel, Switzerland; ^3^Scientific IT Services, ETH ZurichBasel, Switzerland; ^4^Swiss Institute of BioinformaticsBasel, Switzerland; ^5^Department of Microbiology, Nutrition, and Dietetics, Czech University of Life Sciences PraguePrague, Czechia

**Keywords:** *Eubacterium hallii*, trophic interactions, bifidobacterium, fucose, fucosyllactose

## Abstract

Fucosyllactoses (2′- or 3′-FL) account for up to 20% of human milk oligosaccharides (HMOs). Infant bifidobacteria, such as *Bifidobacterium longum* subsp. *infantis*, utilize the lactose moiety to form lactate and acetate, and metabolize L-fucose to 1,2-propanediol (1,2-PD). *Eubacterium hallii* is a common member of the adult gut microbiota that can produce butyrate from lactate and acetate, and convert 1,2-PD to propionate. Recently, a Swiss cohort study identified *E. hallii* as one of the first butyrate producers in the infant gut. However, the global prevalence of *E. hallii* and its role in utilization of HMO degradation intermediates remains unexplored. Fecal 16S rRNA gene libraries (*n* = 857) of humans of all age groups from Venezuela, Malawi, Switzerland, and the USA were screened for the occurrence of *E. hallii*. Single and co-culture experiments of *B. longum* subsp. *infantis* and *E. hallii* were conducted in modified YCFA containing acetate and glucose, L-fucose, or FL. *Bifidobacterium* spp. (*n* = 56) of different origin were screened for the ability to metabolize L-fucose. Relative abundance of *E. hallii* was low (10^−5^–10^−3^%) during the first months but increased and reached adult levels (0.01–10%) at 5–10 years of age in all four populations. In single culture, *B. longum* subsp. *infantis* grew in the presence of all three carbohydrates while *E. hallii* was metabolically active only with glucose. In co-culture *E. hallii* also grew with L-fucose or FL. In co-cultures grown with glucose, acetate, and glucose were consumed and nearly equimolar proportions of formate and butyrate were formed. *B. longum* subsp. *infantis* used L-fucose and produced 1,2-PD, acetate and formate in a ratio of 1:1:1, while 1,2-PD was used by *E. hallii* to form propionate. *E. hallii* consumed acetate, lactate and 1,2-PD released by *B. longum* subsp. *infantis* from FL, and produced butyrate, propionate, and formate. Beside *B. longum* subsp. *infantis, Bifidobacterium breve*, and a strain of *B. longum* subsp. *suis* were able to utilize L-fucose. This study identified a trophic interaction of infant *bifidobacteria* and *E. hallii* during L-fucose degradation, and pointed at *E. hallii* as a metabolically versatile species that occurs in infants and utilizes intermediates of bifidobacterial HMO fermentation.

## Introduction

Human milk oligosaccharides (HMOs) are one of the major glycan source of the infant gut microbiota. Primary components of HMOs are D-glucose, D-galactose, L-fucose, N-acetylglucosamine, and sialic acid. Lactose constitutes the reducing end of HMOs, its galactose moiety can be fucosylated or sialylated to form 2′- or 3′-fucosyllactose (2′-FL or 3′-FL), or 3′- and 6′-sialyl-lactose (3′-SL or 6′-SL). Lactose can also be elongated with units of N-acetyllactosamine (Gal-β1-4GlcNAc) with its simplest form being Lacto-N-neotetraose (LNnT) (Kunz et al., 2000). The composition of HMOs is individually different and remains stable during different lactation phases (Niñonuevo et al., 2008; de Leoz et al., 2012). FLs can account for up to 20% of all HMOs (Niñonuevo et al., 2008; de Leoz et al., 2012).

Infant bifidobacteria species, such as *Bifidobacterium longum* subsp. *infantis* and *Bifidobacterium bifidum*, are adapted to degrade HMOs (LoCascio et al., 2010; Rockova et al., 2012; Turroni et al., 2014) and constitute a big proportion of the infant intestinal microbiota immediately after birth (Avershina et al., 2013; Jost et al., 2014). The degradation of HMOs relies on a complex network of extracellular solute binding proteins, transporters, and intra- or extracellular glycosyl hydrolases (GH). *B. longum* subsp. *infantis* carries out intracellular degradation while *B. bifidum* metabolizes HMOs extracellularly (Garrido et al., 2015). Both *B. longum* subsp. *infantis* and *B. bifidum* possess several α-fucosidases that release L-fucose from FL (Sela et al., 2012; Garrido et al., 2015). L-fucose can then be either used by the strain itself or can be released for bacterial cross-feeding (Garrido et al., 2015; Bunesova et al., 2016).

L-fucose is a desoxyhexose that is a common component of many N- and O-linked glycans and of glycolipids produced by mammalian cells (Becker and Lowe, 2003). L-fucose utilization has been investigated in depth in pathogens such as *Escherichia coli, Shigella* spp. and *Bacillus cereus* (Staib and Fuchs, 2014). These bacteria employ a fucose isomerase FucI, a fucose aldolase FucA, and a fucose kinase FucK to form L-lactaldehyde which can be further metabolized to 1,2-propanediol (1,2-PD). Recently, we could show that strains of *B. longum* subsp. *infantis* and *B. longum* subsp. *suis* metabolize L-fucose to 1,2-PD presumably by a pathway that employs non-phosphorylated intermediates similar to *Campylobacter* and *Xanthomonas* spp. (Bunesova et al., 2016). Genes related to L-fucose degradation were located on two genomic regions, one is part of the HMO degradation cluster H1 of *B. longum* subsp. *infantis* DSM 20088 (LoCascio et al., 2010), while region 2 contained a gene encoding a putative fucose permease (Bunesova et al., 2016).

In the intestine, 1,2-PD is a precursor of propionate by a pathway that employs a glycerol/diol dehydratase as a key enzyme (Reichardt et al., 2014). The frequent detection of genes encoding glycerol/diol dehydratases in fecal metagenomes of adults suggested that 1,2-PD conversion significantly contributes to intestinal propionate formation (Reichardt et al., 2014; Engels et al., 2016). One species with the ability to convert 1,2-PD to propionate is *Eubacterium hallii* which is a common commensal in adults (Engels et al., 2016). *E. hallii* formed similar amounts of propionate in the presence or absence of glucose and did not utilize glucose if 1,2-PD was present (Engels et al., 2016). *E. hallii* can also grow and form butyrate using either glucose, or acetate and lactate as substrates (Duncan et al., 2004).

We hypothesized that a trophic interaction between *E. hallii* and *B. longum* subsp. *infantis* can yield short chain fatty acids (SCFAs) butyrate or propionate from lactate and acetate, or from 1,2-PD, respectively. Both butyrate and propionate are important for gut microbiota/host homeostasis as they interact with the host epithelium and impact the immune system. Butyrate is a main energy source of colonocytes, impacts cell proliferation and differentiation, and lowers the risk of colitis and colorectal cancer (Wong et al., 2006; Plöger et al., 2012). Propionate acts as a precursor for gluconeogenesis in the liver and also impacts cell differentiation with potential health-promoting impact on intestinal inflammation, and cancer development (Reichardt et al., 2014).

*Bifidobacterium* is the predominant genus of the gut microbiota of breast fed infants. Little data exists on the occurrence of *E. hallii* in early life. Recently, a Swiss cohort study identified *E. hallii* as one of the first butyrate producers in the infant gut (Pham et al., 2016). However, the global prevalence of *E. hallii* and its role in the metabolism of L-fucose sourced from HMOs remains unexplored.

It was therefore the aim of this study to investigate the occurrence and abundance of *E. hallii* in populations of different age and origin, and to prove trophic interactions of *E. hallii* and *B. longum* subsp. *infantis* during growth in the presence of glucose, L-fucose, and FL. We also screened further *Bifidobacterium* spp. and strains (*n* = 56) to investigate whether species other *B. longum* subsp. *infantis* or subsp. *suis* are able to metabolize L-fucose.

## Methods

### Bacterial strains and culture conditions

*E. hallii* DSM 3353 obtained from the Deutsche Sammlung von Mikroorganismen und Zellkulturen (DSMZ) was cultivated in modified YCFA medium (mYCFA) containing 30 mM acetate as described by Duncan et al. (2004) with slight modifications (Table 1). All components except L-cysteine-HCL (Sigma-Aldrich) were solubilized in deionized water, and pH was adjusted to pH 7.6 with NaOH. The medium was flushed with CO_2_ and boiled. When the color changed from blue to pink, L-cysteine-HCl (0.01%, w/v) was added. The medium was transferred to Hungate tubes flushed with CO_2_, and tubes were sealed and autoclaved. Stab cultures of *E. hallii* that were frozen at −20°C in mYCFA agar (1.5% (w/v) agar) were used as stock cultures. For each experiment, a fresh agar stock was thawed; 1 ml of liquid YCFA medium was added and thoroughly shaken before being transferred to 8 ml liquid mYCFA medium. After incubation at 37°C for 24 h, the culture was transferred at least once to fresh mYCFA broth before the experiment. *Bifidobacterium* spp. (Table 2) were obtained from the culture collections of the Laboratory of Food Biotechnology of ETH Zurich, the Department of Microbiology, Nutrition, and Dietetics, of the Czech University of Life Sciences Prague, or from DSMZ. Stock cultures of bifidobacteria were maintained at −80°C in 30% glycerol. To prepare working cultures, bifidobacteria were streaked on Wilkens–Chalgren medium (Oxoid) supplied with soya peptone (5 g L^−1^, Biolife, WCSP), Tween 80 (1 mL L^−1^, Sigma-Aldrich), and fresh, filter-sterilized L-cysteine-HCl (0.5 g L^−1^). Single colonies were picked and were grown in liquid WCSP supplied with fresh, filter-sterilized L-cysteine-HCl (0.5 g L^−1^) at 37°C for 24 h. For preparation of co-culture experiments, 100 μL of *B. longum* subsp. *infantis* overnight culture grown liquid WCSP were added to mYCFA medium, and the culture was incubated at 37°C for 24 h. Unless otherwise stated, mYCFA containing 55 mM glucose was used to routinely cultivate *E. hallii* and *B. longum* subsp. *infantis*.

**Table 1:** **mYCFA medium composition**.

**Component**	**Addition**
Amicase	1% (w/v)
Yeast extract	0.25% (w/v)
Sodium bicarbonate	0.5% (w/v)
Glucose (replaced with L-fucose and FL)	1% (w/v)
Mineral solution [3% (w/v) potassium dihydrogen phosphate, 6% (w/v) sodium chloride, 0.6% (w/v) magnesium sulfate, 0.6% calcium chloride (w/v)]	15% (v/v)
Vitamin solution [0.01% (w/v) biotin, 0.01 (w/v) cobalamin, 0.03% p-aminobenzoic acid (w/v), 0.05% folic acid (w/v), 0.15% pyridoxamine (w/v)]	0.1% (v/v)
Volatile fatty acid mix [56.6% (v/v) acetic acid, 20% (v/v) butyric acid, 13.3% (v/v) propionic acid]	0.31% (v/v)
Hemin (0.5 mg ml^−1^)	0.02%
Resazurin (1 mg ml^−1^)	0.1%
L-cysteine hydrochloride monohydrate	0.1%

**Table 2:** **L-fucose utilization and 1,2-PD formation of strains of bifidobacteria**.

**Species**	**Strain code**	**Origin**	**L-fucose utilization**	**1,2-PD formation**
*B. breve*	DSM 20213	Intestine of infant	+	nd
	TPY 10-1	Kenyan infant feces, 6 m	+	+
	BSM 1-2	Kenyan infant feces, 6 m	+	+
	TPY 5-1	Kenyan infant feces, 6 m	+	+
	N4-BM5-i12	Swiss mother breast-milk	+	+
	N4-NF3-i1	Swiss infant feces, 1 w	+	+
	N4-NF3-i3	Swiss infant feces, 1 w	+	+
	N4-NF4-i1	Swiss infant feces, 2 w	+	+
	BR03	Probiotic drops	+	nd
	OL2	Czech infant feces, 10 w	+	nd
	TA1	Czech infant feces, 10 w	+	nd
*B. longum* subsp. *infantis*	DSM 20088	Intestine of infant	+	+
	DSM 20090	Intestine of infant	+	nd
	BRS 8-2	Kenyan infant feces, 6 m	+	nd
	TPY 12-1	Kenyan infant feces, 6 m	+	+
	TPY 8-1	Kenyan infant feces, 6 m	−	−
	BV	BIOPRON probiotic product	+	nd
*B. longum* subsp. *longum*	DSM 20219	Intestine of adult	−	−
	TA2	Czech infant feces, 10 w	−	−
	N2-MF1-i1	Swiss adult feces	−	−
	N18-MF4-i8	Swiss adult feces	−	−
	2ToBifN	Czech infant feces	−	−
	MA2	Czech infant feces	−	−
	BL13	Czech adult feces	−	−
*B. longum* subsp. *suis*	DSM 20211	Pig feces	−	−
	5/9	Calf feces	−	−
	022II	Calf feces	−	−
	BSM 11-5	Kenyan infant feces, 6 m	+	+
*B. kashiwanohense*	DSM 21854	Infant feces, 1.5 y old	−	−
	PV 20-2	Kenyan infant feces, 6 m	−	−
	TPY11-1	Kenyan infant feces, 6 m	−	−
*B. bifidum*	DSM 20456	Feces breast fed infant	−	−
	DSM 20239	Feces breast fed infant	−	−
	DSM 20082	Adult feces	−	−
	DSM 20215	Adult feces	−	−
	BRS 26-2	Kenyan infant feces, 6 m	−	−
	BSM 28-1	Kenyan infant feces, 6 m	−	−
	BRS300	Kenyan infant feces, 6 m	−	−
*B. thermophilum*	RBL67	Infant feces	−	−
*B. animalis* subsp. *lactis*	N1-MF3-i7	Intestine of adult	−	−
*B. adolescentis*	JK3	Czech infant feces, 3 w	−	−
	JK10	Czech adult feces	−	−
	JK17	Czech adult feces	−	−
	1MBif	Czech adult feces	−	−
*B. catenulatum*	DSM 16992	Human feces	−	−
	20ToBifN	Czech infant feces	−	−
	10VoBif	Czech infant feces	−	−
*B. pseudocatenulatum*	N18-NF4-i5	Swiss infant feces, 1 m	−	−
*B. dentium*	DSM 20436	Dental caries	−	−
	VBif10D2	Czech infant feces	−	−
*B. angulatum*	DSM 20098	Human feces	−	−
*B. minimum*	DSM 20102	Sewage	−	−
*B. pseudolongum* subsp. *pseudolongum*	DSM 20099	Pig feces	−	−
	DSM 20095	Chicken feces	−	−
*B. pseudolongum* subsp. *globosum*	DSM 20092	Rumen	−	−
	PV8-2	Kenyan infant feces, 6 m	−	−

*Utilization was tested in API medium supplied with 5 g L^-1^ L-fucose and trace amounts of glucose. Color shift of the API medium after 48 h of incubation indicated L-fucose utilization (+). 1,2-PD formation was verified by HPLC-RI for selected samples (Table 3). nd, not determined; m, months; w, weeks*.

### Single and co-culture studies in the presence of different substrates

Growth kinetics were assessed in mYCFA medium supplied with glucose (50 mM, mYCFA_glc, Sigma-Aldrich), L-fucose (40 mM, mYCFA_fuc, Sigma-Aldrich), or FL (6 mM 2′-FL and 6 mM 3′-FL, mYCFA_FL, Glycom A/S). Trace amounts of glucose were added to mYCFA_fuc to enforce initial growth (Bunesova et al., 2016). Hungate tubes containing 9 ml mYCFA_glc, mYCFA_fuc, or mYFA_FL were inoculated with overnight cultures of *E. hallii* and *B. longum* subsp. *infantis* (0.25 mL each). For comparison, *E. hallii* and *B. longum* subsp. *infantis* were also grown in single cultures. Samples were taken after 0, 4, 8, 12, 24, and 48 h of incubation for substrate and metabolite analysis, and for DNA isolation. Bacterial growth was evaluated by measuring the optical density at 600 nm (OD_600_). Additionally, 16S rRNA gene copies of *E. hallii* and *B. longum* subsp. *infantis* were determined in the co-cultures as outlined below. Growth was investigated in at least independent triplicates with the exception of *B. longum* subsp. *infantis* growth in mYCFA_glc and mYCFA_fuc, which was only investigated in duplicates. Therefore, standard deviations are not shown in the respective graphs.

### Screening of bifidobacteria strains for L-Fucose utilization

Overnight bifidobacteria cultures grown in liquid WCSP were washed and resuspended in phosphate buffered saline (PBS). Bifidobacteria (50 μL) were inoculated in 950 μL API medium supplied fresh, filter-sterilized L-cysteine-HCl (0.5 g L^−1^) and with 30 mM glucose, or with 30 mM L-fucose and trace amounts of glucose. Cultures were incubated at 37°C for 48 h. Growth and utilization of the carbohydrate source was judged by color change of the medium from blue to yellow. For selected strains, L-fucose utilization and metabolite formation were assessed by HLPC-RI as outlined below.

### Analysis of substrate utilization and metabolite formation

Glucose and L-fucose consumption, and the formation of 1,2-PD, lactate, acetate, formate, butyrate, and propionate was measured using high performance liquid chromatography (Merck-Hitachi) equipped with an Aminex HPX-87H column (300 × 7.8 mm; BioRad) and a refractive index detector (HPLC-RI). Samples were centrifuged at 13,000 *g* for 5 min at 4°C. Supernatants (40 μL injection volume) were eluted with 10 mM H_2_SO_4_ at a flow rate of 0.6 ml min^−1^ at 40°C. Sugars, SCFAs, 1,2-PD, and lactate (all Sigma-Aldrich) were quantified using external standards.

Propanal and propanol were quantified with ion chromatography with pulsed amperometric detection (IC-PAD) on a ICS-5000^+^ system (Thermo Scientific) equipped with a quaternary gradient pump, a thermoautosampler, and an electrochemical detector with a cell containing an Ag/AgCl reference electrode and a disposable thin-film platinum working electrode tempered at 25°C. Analytes were separated on a IonPac ICE-AS1 4 × 250 mm ion-exclusion column with guard column (Thermo Scientific) operated at 30°C using isocratic conditions (0.1 M methanesulfonic acid; 0.2 mL min^−1^) for 36 min. The injection volume was 10 μL. Electrochemical data was obtained using a triple potential waveform consisting of regeneration/detection, oxidation, and reduction potentials: *E*_1_ = 0.3 V (*t*_1_ = 0.31 s), *E*_2_ = 1.25 V (*t*_2_ = 0.34 s, *t*_int_ = 0.02 s), *E*_3_ = −0.4 V (*t*_3_ = 0.39 s). Currents were measured and integrated with respect to time (*t*_int_). Propanal and propanol (Sigma-Aldrich) were quantified using external standards.

### DNA isolation and quantification *E. hallii* and *B. longum* subsp. infantis in infant feces and in co-cultivation studies

Genomic DNA was isolated from 0.5 mL fermented mYCFA using the FastDNA SPIN Kit for Soil (MP Biomedicals). Genomic DNA from stool samples (*n* = 368) collected as part of an infant cohort study, and of Swiss children and adult had been isolated as described before (Vanderhaeghen et al., 2015; Pham et al., 2016). The abundance of *E. hallii* was determined using primers EhalF (5′- GCGTAGGTGGCAGTGCAA -3′) and EhalR (5′- GCACCGRAGCCTATACGG-3′) (Ramirez-Farias et al., 2009). *B. longum* subsp. *infantis* was quantified using primer pair F (5′-TCGCGTCYGGTGTGAAAG-3′), and R (5′-CCACATCCAGCRTCCAC-3′) (Rinttilä et al., 2004). Primers Eub338F (5′-ACTCCTACGGGAGGCAGCAG-3′) and Eub518R (5′- ATTACCGCGGCTGCTGG-3′) were employed to quantify total bacteria 16S rRNA genes (Fierer et al., 2005). Reactions were performed using a 7500 Fast Real-Time PCR System (Applied Biosystems) and the Kapa SYBR FAST qPCR mastermix (Biolab Scientifics Instruments SA). Thermal cycling started with an initial denaturation step at 95°C for 3 min, followed by 40 cycles consisting of denaturation at 95°C for 3 s, annealing at 60°C for 10 s, and elongation at 72°C for 25 s. To verify specificity of amplification, melting curve analysis and agarose gel electrophoresis for amplicon size control were performed. To generate standards, PCR amplicons were cloned into PGEMT Easy Vector and heterologously expressed in *E. coli* according to instructions of the supplier (Promega). Standard curves were prepared from ten-fold dilutions of linearized plasmids harboring the 16S rRNA gene of interest. Linear detection range was between, log 2.3 and log 8.3 gene copies for *E. hallii* 16S rRNA genes, between log 2.9 and log 8.9 gene copies bifidobacteria 16S rRNA genes, and between log 3.0 and log 8.0 gene copies for total bacteria 16S rRNA genes. A factor of 5.5 and 4 for *Eubacterium* spp. and *B. longum* subsp. *infantis*, (rrnDB, http://rrndb.mmg.msu.edu; Větrovský and Baldrian, 2013), respectively, was applied to calculate the numbers of cells accounting for several 16S rRNA gene copies per genome.

### 16s rRNA gene amplicon libraries screens

16S rRNA gene sequencing datasets published by Yatsunenko et al. (2012) were downloaded from MG-RAST (MG-RAST ID 401). The 489 datasets with known age of the donor contained in total 1.0^*^10^9^ sequences and on average 2.1^*^10^6^ sequences. All reads were aligned against the Silva database (version 123.1, Quast et al., 2013) using MALT in semiglobal alignment mode (Herbig et al., 2016) and only matches with a percent identity >97% were reported. Alignments were then used to assign reads on the Silva taxonomy. A read was placed on the lowest taxon so that at least 90% of the alignments were covered by that taxon (majority vote 90%). Unaligned reads were extracted and placed on the Silva taxonomy by using the rdp classifier with a cutoff of 0.8.

### Identification of L-Fucose utilization related genes in *B. breve* genomes

Genomes of *Bifidobacterium breve* DSM 20213 (PRJDB57) and UCC 2003 (CP000303.1) were screened for genes encoding proteins related to L-fucose metabolism using BlastP and the corresponding proteins of *B. longum* subsp. *infantis* DSM 20088 region 1 and 2 for the query (Bunesova et al., 2016, see also below).

### Screening of shotgun sequencing datasets for presence of proteins encoding L-Fucose utilization regions 1 and 2

Metagenomic datasets (*n* = 111) published by Yatsunenko et al. (2012) were downloaded from MG-RAST (MG-RAST ID 98). The datasets contained in total 16,318,166 sequences and on average 147,010 sequences per sample. The smallest and largest datasets contained 19,587 and 478,588 sequences, respectively, with an average sequence length of 358 bases. All datasets were aligned using DIAMOND blastx in sensitive mode (Buchfink et al., 2015) against a modified bacterial RefSeq database (Pruitt et al., 2007). The modified RefSeq database was composed by adding 24 protein sequences of *B. longum* subsp. *infantis* DSM 20088 and *B. breve* DSM 20213 fucose utilization regions 1 and 2 (Bunesova et al., 2016, WP_003830405.1, WP_003830403.1, WP_003830401.1, WP_003830400.1, WP_065457149.1, WP_014484327.1, WP_003829769.1, WP_003829768.1, WP_025300063.1, WP_013141362.1, WP_003829764.1, WP_012578562.1, WP_012578563.1, WP_012578564.1, WP_012578565.1, WP_012578566.1, WP_012578567.1, WP_012578568.1, WP_013141357.1, WP_012578533.1, WP_012578534.1, WP_012578535.1, WP_012578536.1, WP_012578537.1) to the standard RefSeq database. Reads were assigned to the best aligning protein sequences which had a bitscore >50. Reads assigned to the 24 proteins sequences of interest were reported.

### Statistical analysis

A sigmoidal non-linear regression model [Weibull, 5 Parameter model, f = if(x < = x0-b^*^ln(2)^∧^(1/c); y0; y0+a^*^(1-exp(-(abs(x-x0+b^*^ln(2)^∧^(1/c))/b)^∧^c))) implemented in SigmaPlot 13, Systat Software] was chosen to fit curves to log% abundance data of *E. hallii* in the different populations. Student's paired *t*-test with two-tailed distribution was used to identify significant differences in metabolite formation, OD_600 nm_, and cell counts between treatments. A *p* < 0.05 was considered significant.

## Results

### Age and geographical-dependent occurrence of *E. hallii*

**Figure 1:**
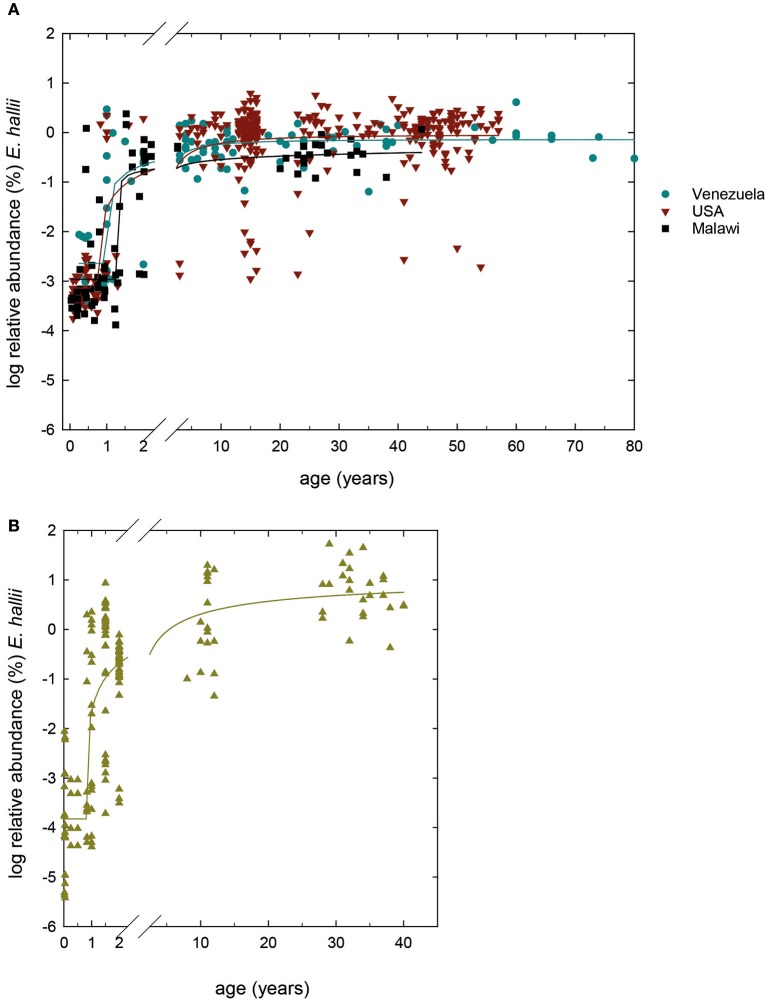
**Global occurrence of ***E. hallii***. (A)** Log % abundance of *E. hallii* in populations from USA, Malawi and Venezuela extracted from 16S rRNA gene amplicon libraries (Yatsunenko et al., 2012) (*n* = 489). **(B)** Log% abundance of *E. hallii* in feces of donors of different age groups from Switzerland (*n* = 348). A sigmoidal non-linear regression model was chosen to fit curves to log % abundance data of *E. hallii* in the different populations.

We screened 489 previously obtained 16S rRNA gene amplicon libraries from Malawi, Venezuela and USA (Yatsunenko et al., 2012) for the occurrence of *E. hallii*. Concurrently, we determined *E. hallii* relative abundance in a Swiss infant cohort study which followed the fecal microbiota of 40 infants for the first 2 years of life (Pham et al., 2016) using qPCR, and compared to Swiss teenagers and Swiss adults (Vanderhaeghen et al., 2015; together *n* = 368; Figure 1). Minimum detection limits were ~10^−4^ and 10^−5^% relative abundance for 16S rRNA gene libraries and qPCR screenings, respectively.

In populations from Venezuela, the USA, and Malawi, *E. hallii* occurred in all infant samples of 1 year of age or younger at means of log −2.0 ± 1.2, −2.9 ± 1.0, and −2.9 ± 0.8%, respectively (Figure 1A). Relative abundance increased after 1–1.5 years in donors from Venezuela (100% occurrence), the USA (95% occurrence), and Malawi (100%). In Swiss infants, occurrence levels of *E. hallii* fluctuated between 13 and 40% until 1 year, and increased to 85% at 2 years of age (log −2.9 ± 0.8%; Figure 1B). Between 5 and 10 years, relative abundance of *E. hallii* reached adult levels in all four populations (Malawi: log −0.4 ± 0.3%, Venezuela: log −0.2 ± 0.3%, USA: log −0.1 ± 0.6%, Switzerland: log 0.8 ± 0.5%).

### Growth of *E. hallii* and *B. longum* subsp. *infantis* in single- and co-cultures

To investigate trophic interactions between *B. longum* subsp. *infantis* and *E. hallii* in the presence of glucose, L-fucose and FL, *B. longum* subsp. *infantis*, and *E. hallii* were grown in single- and co-cultures in mYCFA.

**Figure 2:**
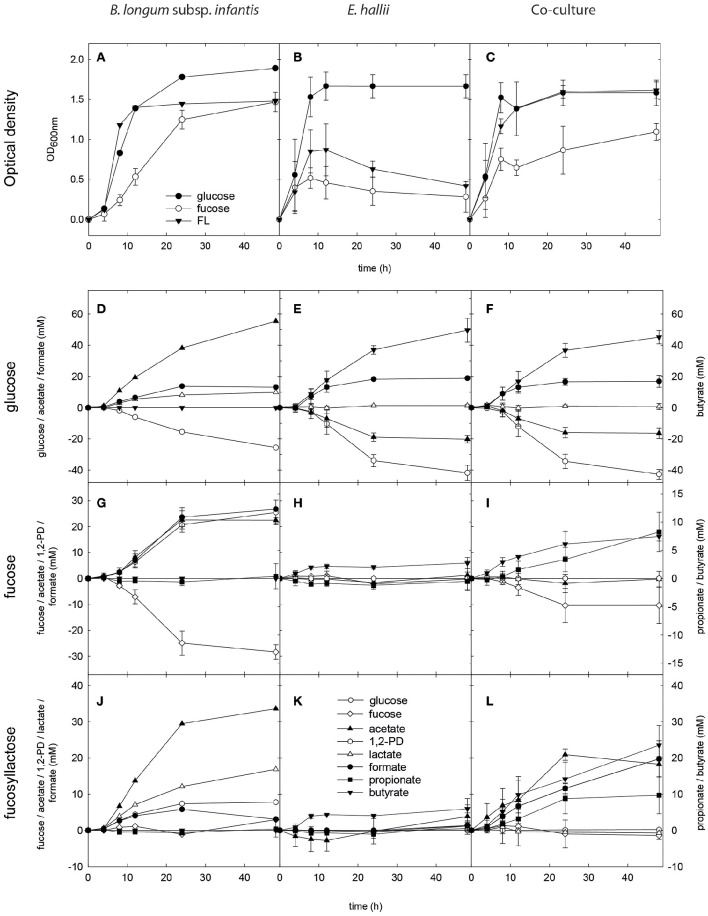
**Growth, substrate utilization, and metabolite formation of ***E. hallii*** and ***B. longum*** subsp. ***infantis*** in single and co-culture**. Optical density (OD_600nm_) of *B. longum* subsp. *infantis* **(A)**, *E. hallii* **(B)**, and the *B. longum* subsp. *infantis* / *E. hallii* co-culture **(C)** during growth in modified YCFA supplied with glucose, L-fucose or FL. Substrate utilization and metabolite formation of *B. longum* subsp. *infantis* **(D,G,J)**, *E. hallii* **(E,H,K)**, and the *B. longum* subsp. *infantis* / *E. hallii* co-culture **(F,I,L)** in mYCFA supplied with glucose **(D–F)**, L-fucose **(G–I)**, or FL **(J–L)**.

In single culture, *B. longum* subsp. *infantis* reached a final optical density (OD_600 nm_ 1.9) in the presence of glucose, and a lower maximal OD_600 nm_ when grown with L-fucose and FL (1.5 ± 0.1 and 1.5, respectively) after 48 h of incubation (Figure 2A). In mYCFA_glc −25.6 mM glucose was used, and 55.4, 9.9, and 13.1 mM acetate, lactate, and formate were formed (Figure 2D). In mYCFA_fuc, *B. longum* subsp. *infantis* used −28.3 ± 2.8 mM L-fucose and produced nearly equimolar amounts of 1,2-PD, acetate and formate (25.5 ± 4.8, 22.4 ± 1.0, and 26.7 ± 0.5 mM, respectively; Figure 2G). Lactate was produced at low amounts (2.0 ± 1.0 mM), and mean carbon recovery was 86%. In the presence of FL, *B. longum* subsp. *infantis* formed acetate (33.6 mM), lactate (16.8 mM), 1,2-PD (7.8 mM), and low amounts of formate (3.1 mM; Figure 2J).

In single culture, *E. hallii* grew rapidly to OD_600 *nm*_ of 1.7 ± 0.2 after 12 h of incubation in mYCFA_glc (Figure 2B). From 1 mol glucose and 0.5 mol acetate, ~1 mol butyrate and 0.5 mol formate were produced (Figure 2E). Maximum optical density in mYCFA_FL and mYCFA_fuc was significantly lower than with glucose with OD_600 nm_ 0.9 and 0.5 after 12 and 9 h of incubation, respectively (Figure 2B). With L-fucose and FL, *E. hallii* formed 2.7 ± 1.0 and 6.0 ± 1.1 mM butyrate, respectively (Figures 2H,K). No acetate utilization and formate production were detected.

**Figure 3:**
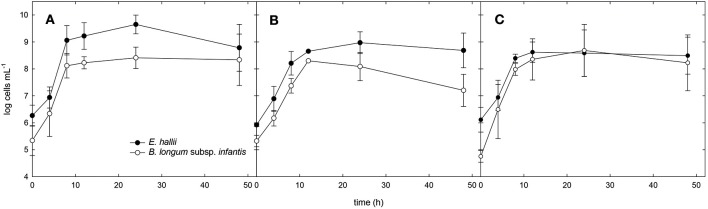
**Cell counts of ***B. longum*** subsp. ***infantis*** and ***E. hallii*** during growth in co-culture**. Cell counts of *B. longum* subsp. *infantis* and *E. hallii* during growth in co-culture in mYCFA_glc **(A)**, mYCFA_fuc **(B)**, and mYCFA_FL **(C)**.

For the co-cultures, qPCR was used in addition to optical density measurements to monitor the growth of both strains (Figure 3). Cell counts were calculated based on 16S rRNA genes corrected for several 16S rRNA gene copies per genome. In mYCFA_glc, cell counts of both strains increased by log 2.8 cells ml^−1^ during the first 8 h of incubation, after which growth slowed down (Figure 3A). Highest OD_600 nm_ was reached after 24 h of incubation (Figure 2C). The amount of acetate used, and butyrate and formate formed in the co-cultures was similar to *E. hallii* single cultures (Figure 2F), and only low amounts of lactate (0.6 ± 0.7 mM) were detected after 48 h (Figure 2F).

Co-cultures grown in mYCFA_fuc reached a maximum OD of 1.1 ± 0.1 after 48 h of incubation (Figure 2C). During the first 12 h, *B. longum* subsp. *infantis* and *E. hallii* grew exponentially with increased cell counts of log 3 and log 2.7 cells mL^−1^, respectively (Figure 3B). In co-cultures, *B. longum* subsp. *infantis* utilized only ~35% of L-fucose compared to single cultures (10.4 ± 8 mM). The co-cultures formed propionate (8.2 ± 3.5 mM), butyrate (7.4 ± 0.8 mM), and formate (10.9 ± 1.7 mM; Figure 2I). Butyrate formed by the co-cultures was significantly (*p* < 0.05) higher compared to butyrate formation of *E. hallii* single culture in mYCFA_fuc (7.4 ± 0.8 vs. 2.7 ± 1.0 mM). 1,2-PD, propanal, and propanol were not detected during co-culture fermentations, and there was no apparent accumulation or consumption of acetate or lactate (Figure 2I).

When grown in co-cultures in mYCFA_FL, cell counts of *B. longum* subsp. *infantis* and *E. hallii* increased by 3.2 and 2.8 log during the first 8 h of incubation (Figure 3C), and maximal OD was reached after 24 h of incubation (Figure 2C). Acetate (18.2 ± 1.6 mM), propionate (9.7 ± 5.1 mM), and butyrate (5.4 ± 3.1 mM), and formate (19.7 ± 5.0 mM) were detected after 48 h of incubation (Figure 2L). 1,2-PD and lactate were only present at low levels <1 mM.

### *Bifidobacterium* production of 1,2-PD from L-fucose

To investigate whether L-fucose utilization was a trait limited to *B. longum* subsp. *infantis*, or was also present in other infant-, adult-, or animal-associated bifidobacteria, we screened *n* = 56 *Bifidobacterium* strains (Table 2) for growth in API medium supplied with 30 mM L-fucose and trace amounts of glucose. All isolates were capable of growing in API medium supplied with 30 mM glucose indicating the suitability of the assay. Only strains of *B. longum* subsp. *infantis* and subsp. *suis*, and of *B. breve* were identified as being able to metabolize L-fucose and to form 1,2-PD. L-fucose was used and 1,2-PD was formed in a ratio of ~1:1 by all L-fucose-utilizing strains (Table 3). Additionally, lactate, acetate, and formate were produced leading to carbon recoveries between 87 and 109% (Table 3).

**Table 3:** **Substrate utilization, metabolite formation and carbon recovery**.

		**Substrate consumption (mM)**	**Metabolite production (mM)**				**Carbon recovery (%)**
**Species**	**ID**	**L-fucose**	**Lactate**	**Formate**	**Acetate**	**1,2-PD**	
*B. breve*	TPY 10-1	−14.9 ± 4.5	10.2 ± 5.2	6.3 ± 6.0	9.2 ± 6.7	13.8 ± 4.0	109
	BSM 1-2	−8.5 ± 1.1	5.3 ± 4.4	4.2 ± 3.0	3.1 ± 2.0	9.3 ± 1.3	105
	TPY 5-1	−12.7 ± 0.5	6.5 ± 2.3	5.4 ± 0.9	6.5 ± 4.1	11.4 ± 1.4	94
	N4-BM5-i12	−8.3 ± 3.9	4.6 ± 2.5	4.0 ± 0.5	2.5 ± 3.4	6.8 ± 1.9	87
	N4-NF3-i1	−11.7 ± 3.1	7.2 ± 1.5	6.6 ± 6.7	5.5 ± 5.0	11.2 ± 2.8	99
	N4-NF3-i3	−5.9 ± 1.4	6.1 ± 1.5	0.6 ± 1.1	1.4 ± 0.9	5.7 ± 1.7	102
*B. longum* subsp. *infantis*	DSM 20088	−3.9 ± 0.7	3.8 ± 0.4	−0.1 ± 0.2	0.8 ± 1.4	3.9 ± 0.8	109
	TPY 12-1	−3.8 ± 1.5	1.4 ± 0.5	1.2 ± 1.1	1.6 ± 0.3	3.8 ± 1.1	91
*B. longum* subsp. *Suis*	BSM 11-5	−11.5 ± 1.0	5.8 ± 1.4	4.8 ± 4.2	3.3 ± 0.9	11.2 ± 1.3	91

*L-fucose utilization and lactate, formate, acetate, and 1,2-PD formation of selected bifidobacteria in API medium supplied with 30 mM L-fucose and trace amounts of glucose after 48 h of incubation at 37°C*.

### Genes related to L-fucose utilization in *B. breve* genomes

**Figure 4:**
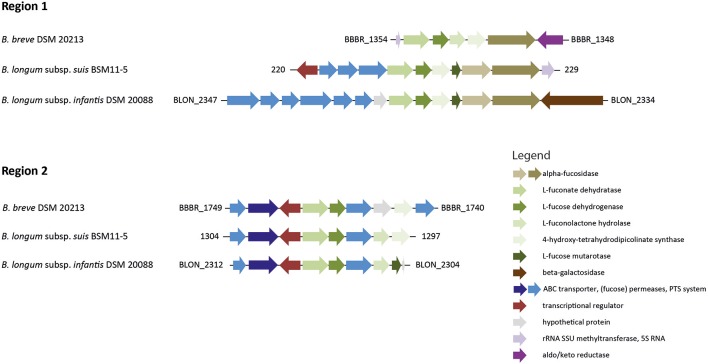
**Genomic regions encompassing genes putatively involved in L-fucose degradation in ***B. longum*** and ***B. breve*****. The gene cluster of region 1 also contained genes encoding the α-fucosidases BLON_2335 and BLON_2336 and is part of the *B. longum* subsp. *infantis* HMO utilization operon H1 (adapted from LoCascio et al., 2010; Bunesova et al., 2016 not drawn according to scale).

We previously identified two genomic regions that encompass genes potentially involved in L-fucose utilization of strains of *B. longum* subsp. *infantis* and *B. longum* subsp. *suis* (Bunesova et al., 2016). The genome of *B. breve* DSM 20213 (PRJDB57) possessed a set of genes with slightly differing genome organization to *B. longum* subsp. *infantis* and *B. longum* subsp. *suis* (Figure 4). Region 1 encompassed a gene encoding an α-fucosidase (GH family 95) with 78% homology in 607/783 AA to BLON_2335 of *B. longum* subsp. *infantis* DSM 20088. Region 1 also contained genes encoding a putative L-fuconate dehydratase (96% homology in 284/293 AA to *B. longum* subsp. *infantis* DSM 20088 L-fuconate dehydratase, region 1), a L-fucose dehydrogenase (93% homology in 245/262 AA, region 1), L-fuconolactone hydrolase (50% homology in 132/264 AA, region 2), and a 4-hydroxy-tetrahydrodipicolinate synthase (89% homology in 265/296 AA, region 1). *B. breve* region 2 contained another L-fuconate dehydratase (97% homology in 417/426 AA, region 2), L-fucose dehydrogenase (94% homology in 249/263 AA, region 2), a putative fucose permease (62% homology in 262/461 AA, region 2), a L-fuconolactone hydrolase (96% homology in 246/255 AA, region 2), and a 4-hydroxy-tetrahydrodipicolinate synthase (69% homology in 206/296 AA, region 1; Figure 4). *B. breve* UCC 2003 had an identical genomic set-up to *B. breve* DSM 20213 (data not shown).

### Coding potential for bifidobacteria fucose utilization in fecal metagenomes

**Figure 5:**
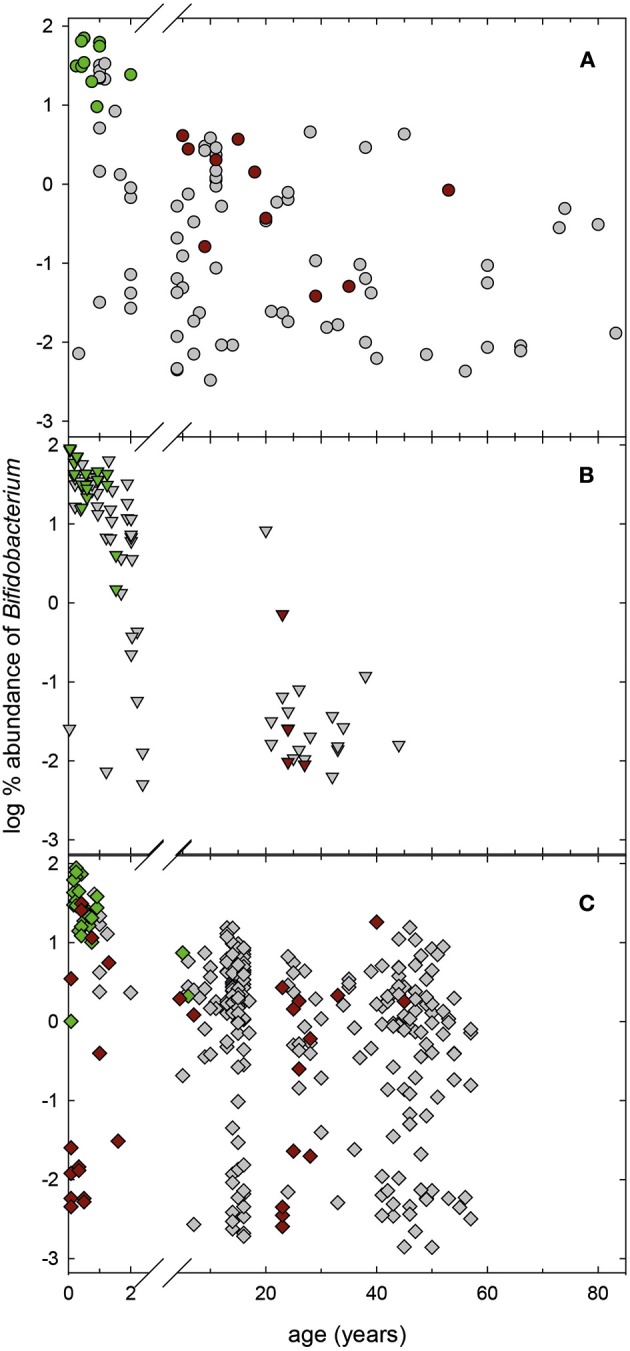
**Relative abundance of the genus ***Bifidobacterium*** in infant fecal samples**. Relative abundance of *Bifidobacterium* in 16S rRNA gene libraries of infant feces collected from Venezuela **(A**, *n* = 98**)**, Malawi **(B**, *n* = 84**)**, and USA **(C**, *n* = 307; Yatsunenko et al., 2012**)**. For samples with available metagenomes (Yatsunenko et al., 2012), the presence of genes encoding bifidobacterial L-fucose utilization related proteins. Green symbols, samples that were positive for bifidobacteria L-fucose utilization related proteins, red symbols, samples that were negative, gray samples, no metagenomes available.

Bifidobacteria were the predominant taxa in feces of infants from Venezuela, Malawi, and the USA during the first 2 years of life based on 16S rRNA gene sequencing data (Figure 5). For 111 samples, metagenomic data of the same donor data were also available (Yatsunenko et al., 2012). Metagenomes were screened for the presence of proteins presumably involved in bifidobacterial L-fucose utilization (Figure 4). At the threshold of detection allowed by sequencing coverage, the majority of fecal metagenomes of infants under 2 years of all three populations possessed the coding potential for bifidobacterial fucose utilization (Figure 5). All infant metagenomes from Venezuela (*n* = 11, 0.25–2 years) had coding potential for bifidobacteria L-fucose utilization. Eighty-seven and Seventy-one percent of proteins of both regions were recovered for *B. longum* subsp. *infantis* and *B. breve* respectively; all infants were positive for *B. longum* subsp. *infantis* while *B. breve* L-fucose utilization was not detected in 1 infant. Similarly, all infant metagenomes from Malawi (*n* = 18, 0.05–1.53 years) had coding potential for bifidobacteria L-fucose utilization. On average, 93 and 74% of the proteins of *B. longum* subsp. *infantis* and *B. breve* were detected, respectively. Of the 44 fecal metagenomes of US American infants (0.08–1.6 years), 16 and 28 were negative and positive for bifidobacteria L-fucose utilization, respectively. *B. breve* assigned proteins were detected in all children while *B. longum* subsp. *infantis* L-fucose utilization related proteins were not detected in one infant. The recovery of proteins of region 1 and 2 was 47 and 23% for *B. longum* subsp. *infantis* and *B. breve*, respectively. Bifidobacterial L-fucose utilization related proteins were also detected in feces of a 5 and a 6 year old US American child (Figure 5).

## Discussion

*E. hallii* is a metabolically versatile species that can contribute to intestinal butyrate and propionate formation (Duncan et al., 2004; Engels et al., 2016). In adults, *E. hallii* is a regular constituent of the gut microbiota (Engels et al., 2016). As shown in this study, *E. hallii* persistently occurred in the first months after birth at low abundance of the fecal microbiota and reached adult levels at ~5–10 years of age independent of geographical donor origin. Thus, *E. hallii* is a commensal occurring very early in life which might contribute to metabolic interactions starting at 1–2 years of age when abundance markedly increased in all populations.

**Figure 6:**
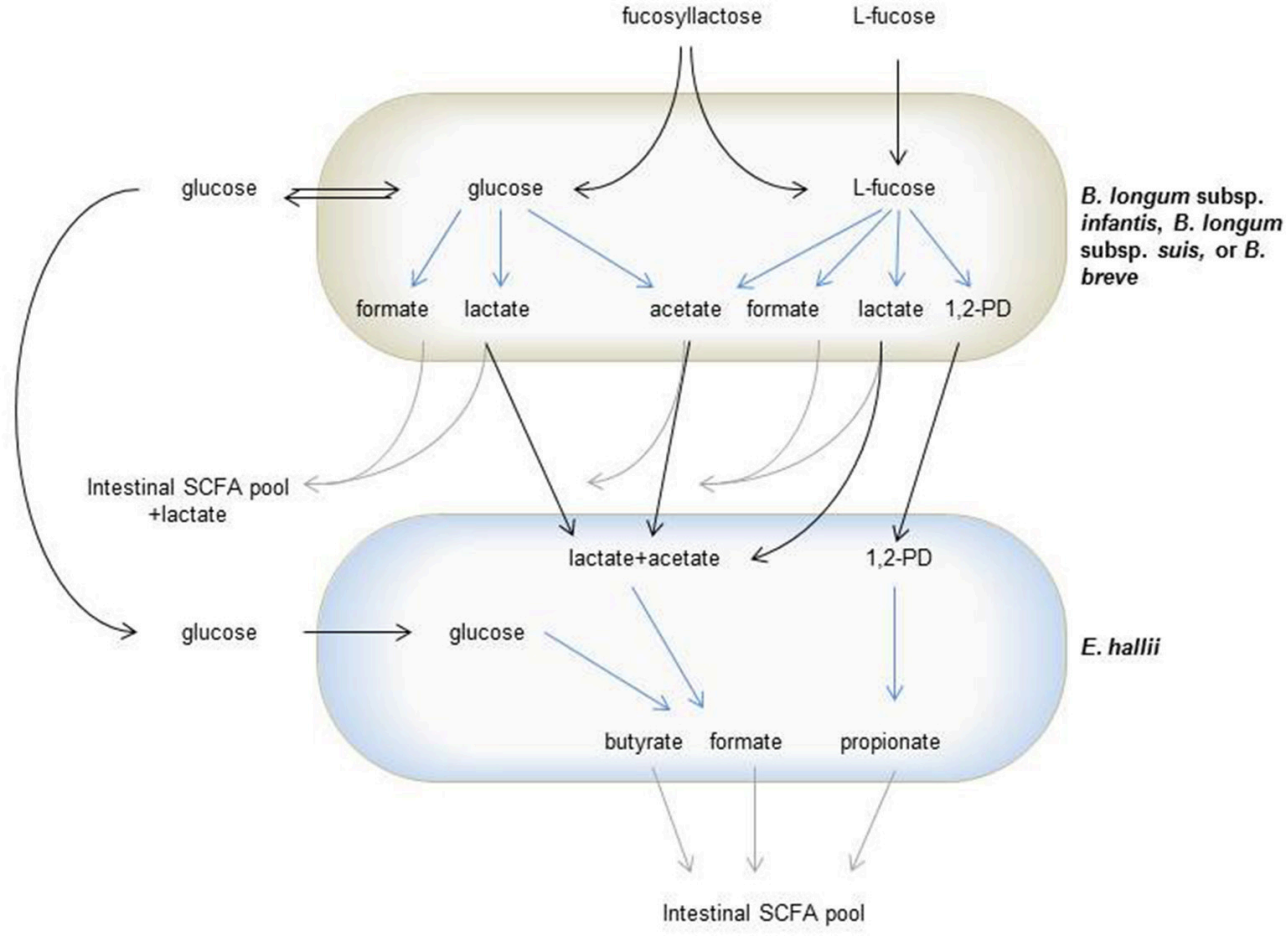
**Trophic interactions of L-fucose utilizing bifidobacteria and ***E. hallii*** during degradation of glucose, L-fucose or FL**. Gray arrows indicate glucose, L-fucose or FL derived degradation metabolites fed in the intestinal SCFA pool, black arrows indicate sugars or metabolites cross-fed between bifidobacteria and *E. hallii*, blue arrows indicate metabolite formation.

*B. breve* and *B. infantis* subsp. *longum* were identified as species capable of metabolizing fucose. Both are two of the most representative species found in breast-milk fed infants (Avershina et al., 2013; Matsuki et al., 2016), and fecal metagenome analysis indicated the coding potential for bifidobacterial fucose utilization in children under 2 years. Trophic interactions of *E. hallii* and *B. longum* subsp. *infantis* and/or *B. breve* during L-fucose and FL utilization can therefore be considered infant specific (Figure 6). Interestingly, general occurrence and the presence of bifidobacteria capable of L-fucose utilization appeared to be higher in Venezuelan and Malawian than in American infants. This might reflect differences in feeding practice, as in Venezuela and Malawi, all analyzed infants below 2 years were breast-fed, while in the USA, 71% received formula (Yatsunenko et al., 2012).

In the infant gut, *E. hallii* can utilize lactate and acetate produced by bifidobacteria during the degradation of hexoses. Bifidobacteria metabolize hexoses via the “bifid shunt” with fructose-6-phosphoketolase as the key enzyme. Glucose (1 mol) theoretically yields 1.5 mol acetate, 1 mol lactate, and 2.5 ATP (de Vries and Stouthamer, 1967, 1968). However, this ratio depends on whether the intermediate pyruvate is cleaved to acetyl phosphate and formate, or whether it is reduced to lactate (Palframan et al., 2003). *B. longum* subsp. *infantis, B. longum* subsp. *suis*, and *B. breve* also metabolized the desoxyhexose L-fucose. In mYCFA, nearly equimolar proportions of acetate, formate and 1,2-PD and only little lactate were formed while in API medium, lactate was produced at the expense of formate and acetate. The ratio of acetate, formate, and lactate formed varies for species, substrate source and carbohydrate supply (limitation or excess; Macfarlane and Gibson, 1995; Palframan et al., 2003). Here, ratios differed for the same species when supplied with the same carbohydrate (30 mM L-fucose). In this study, mYCFA was prepared strict anaerobically while API medium was only facultative anaerobic, which could have also impacted pyruvate metabolism. It was shown before that the presence of oxygen changed final metabolites formed by *B. longum subsp. infantis* (González et al., 2004).

Both bifidobacteria and *E. hallii* were able to produce formate during the degradation of FL (Figure 6). Methanogens, which can produce methane from formate and CO_2_, are usually not detected in infants (Vanderhaeghen et al., 2015). As feces collected as part of a cohort study following 16 infants from 2 weeks to 2 years of age (Pham et al., 2016, unpublished data) contained no or only very low levels of formate, it can be assumed that the formate produced during HMO degradation is further utilized. Formate together with CO_2_ can also be used by acetogenic microbes such as *Blautia* spp. to produce acetate via the Wood-Ljungdal pathway. Little literature exists on formate cross-feeding within the infant gut microbiota. However, as one of the important intermediate metabolites, the effect of formate and formate utilization on infant gut health should be investigated in further studies.

We identified for the first time the ability of *B. breve* strains to metabolize L-fucose to 1,2-PD. We previously suggested a *Bifidobacterium* L-fucose utilization pathway based on genome comparison which has been also identified by a recent study investigating FL degradation by a strain of *B. longum* subsp. *longum* (Bunesova et al., 2016; Garrido et al., 2016). Similar to strains of *B. longum* subsp. *infantis* and *B. longum* subsp. *suis, B. breve* harbored two genomic regions which encompassed genes putatively encoding enzymes involved in L-fucose degradation with non-phosphorylated intermediates. In contrast, the L-fucose-negative *B. kashiwanohense* only possessed region 1 (Bunesova et al., 2016), suggesting that both regions are necessary for L-fucose metabolism. In addition, two of the identified genes encoding fucose permease (FucP) and a 4-hydroxy-tetrahydrodipicolinate synthase, were upregulated when *B. breve* was grown in co-culture with mucin degrading *B. bifidum* (Egan et al., 2014). *B. bifidum* releases L-fucose during growth with mucin similar to the release of fucose from FL (Turroni et al., 2014; Garrido et al., 2015; Bunesova et al., 2016), which then can be imported and metabolized by other species such as *B. breve* or L-fucose-utilizing strains of *B. longum*.

In adults, *B. adolescentis* is a predominant *Bifidobacterium* species, and cross-feeding between *B. adolescentis* and *E. hallii* has been reported before (Belenguer et al., 2006). *E. hallii* was thereby able to form butyrate from lactate and acetate that was produced when *B. adolescentis* grew in the presence of starch, or to use mono- or dissacharides released by *B. adolescentis* from fructooligosaccharides. We also observed substrate-dependent routes of metabolic cross-feeding. In the presence of L-fucose, propionate was the main metabolite formed by *E. hallii*. In co-cultures with *B. longum* subsp. *infantis*, the formation of propionate, butyrate, and formate from FL was observed. Here, butyrate and formate could have been produced from lactate and acetate, or directly from glucose released by *B. longum* subsp. *infantis*, while propionate again was derived from L-fucose.

FL is a major component of HMOs (de Leoz et al., 2012). Therefore, it can be implied that a substantial proportion of L-fucose is metabolized to propionate if *E. hallii* is present. However, not all women are able to secrete α-(1-2)-fucosylated HMOs due to mutations that render the responsible fucosyltransferase FUT2 inactive. About 20% of the European and African population carry an inactive FUT2 (Kelly et al., 1995; Liu et al., 1998). The activity of FUT2 and with that the presence of fucosylated HMOs has been linked to differences in the establishment of the infant gut microbiota (Lewis et al., 2015). The bifidobacteria community of infants born to non-secretor mothers was reported to establish later, to carry reduced numbers of bifidobacteria, and to contain a lower percentage of bifidobacteria capable of degrading 2-FL compared to infants of secretor mothers (Lewis et al., 2015). Thus, non-secretor mothers' milk lacks a significant source for intestinal propionate formation. It could therefore be speculated that the SCFA profile of infants of secretor- and non-secretor mothers is different. However, to date, no large scale study compared the fecal SCFA profile of infants born to secretor and non-secretor mothers.

Fucosylated oligosaccharides are not only a carbon source for the intestinal microbes but also play an important role in host-microbe interactions (Pickard et al., 2014). On one hand, fucosylated HMOs protect against invasion of enteric pathogens acting as a decoy for epithelial attachment sites (Morrow et al., 2005). On the other hand, L-fucose released from fucosylated oligosaccharides has been related to virulence and host colonization of enteric pathogens. It was suggested that L-fucose released by *Bacteroides thetaiotamicron* was utilized by *Salmonella enterica* serovar Typhimurium during colonization of a mouse model (Ng et al., 2013). Likewise, L-fucose released by *B. thetaiotamicron* increased colonization of an enterohaemorrhagic *E. coli* (EHEC) in comparison to mutants with a knock-out of a fucose sensing two-component system (Pacheco et al., 2012). This fucose sensing two-component system regulates virulence and metabolic gene expression of EHEC (Pacheco et al., 2012). The ability to utilize L-fucose provided *Campylobacter jejuni* with a competitive advantage during the colonization of birds or pigs compared to fucose-negative mutants (Muraoka and Zhang, 2011; Stahl et al., 2011). Through utilization of L-fucose, the infant bifidobacterial community therefore could enhance colonization resistance toward enteric pathogens.

## Conclusion

L-fucose utilization was identified here as a trait of only infant-derived bifidobacteria. Trophic interactions of L-fucose-utilizing infant bifidobacteria and *E. hallii* yielded different SCFAs from L-fucose or FL pointing at *E. hallii* as a metabolically versatile species utilizing intermediate metabolites of HMO fermentation. The utilization of L-fucose by the infant bifidobacterial community might enhance colonization resistance toward fucose-dependent enteric pathogens.

## Author contributions

VB and CS designed experiments; VB, CS, VTP, and HR conducted experimental work; VB, CS, VTP, and H-JR analyzed data; CL, NB provided financial support; CS wrote manuscript with the help of all authors.

## Funding

VB was supported by SCIEX grant 13.151. VTP and this research were supported by a grant of the Swiss National Science Fond (PN 310030_146784). H-JR was funded by the European Research Council (ERC Synergy Grant No. 609883 to NB).

### Conflict of interest statement

The authors declare that the research was conducted in the absence of any commercial or financial relationships that could be construed as a potential conflict of interest.
